# Additive manufacturing of laminar flow cells for single-molecule experiments

**DOI:** 10.1038/s41598-019-53151-z

**Published:** 2019-11-14

**Authors:** Arash Ahmadi, Katharina Till, Yngve Hafting, Mark Schüttpelz, Magnar Bjørås, Kyrre Glette, Jim Tørresen, Alexander D. Rowe, Bjørn Dalhus

**Affiliations:** 1 Department of Medical Biochemistry, Institute for Clinical Medicine, University of Oslo, Oslo, Norway; 2 Biomolecular Photonics, Department of Physics, University of Bielefeld, Bielefeld, Germany; 3 Department of Informatics, University of Oslo, Oslo, Norway; 4 Department of Clinical and Molecular Medicine, Faculty of Medicine and Health Sciences, Norwegian University of Science and Technology (NTNU), Trondheim, Norway; 5 Department of Microbiology, Oslo University Hospital HF, Rikshospitalet and University of Oslo, Oslo, Norway; 6 Department of Newborn Screening, Division of Child and Adolescent Medicine, Oslo University Hospital, Oslo, Norway

**Keywords:** Lab-on-a-chip, Single-molecule biophysics

## Abstract

A microfluidic laminar flow cell (LFC) forms an indispensable component in single-molecule experiments, enabling different substances to be delivered directly to the point under observation and thereby tightly controlling the biochemical environment immediately surrounding single molecules. Despite substantial progress in the production of such components, the process remains relatively inefficient, inaccurate and time-consuming. Here we address challenges and limitations in the routines, materials and the designs that have been commonly employed in the field, and introduce a new generation of LFCs designed for single-molecule experiments and assembled using additive manufacturing. We present single- and multi-channel, as well as reservoir-based LFCs produced by 3D printing to perform single-molecule experiments. Using these flow cells along with optical tweezers, we show compatibility with single-molecule experiments including the isolation and manipulation of single DNA molecules either attached to the surface of a coverslip or as freely movable DNA dumbbells, as well as direct observation of protein-DNA interactions. Using additive manufacturing to produce LFCs with versatility of design and ease of production allow experimentalists to optimize the flow cells to their biological experiments and provide considerable potential for performing multi-component single-molecule experiments.

## Introduction

The ever-growing demand for exploration of the micro- and nano-scale world, and its contribution to our current understanding in biology, have played a central role in developments in the field of microscopy. Observing biological processes at the single molecule level, in controlled settings, reveals an unprecedented level of detail and crucial aspects of the molecular mechanisms that would not be observed in bulk-scale experiments. Understanding these mechanisms at the cellular and molecular level provides a deeper insight into the diversity and range of functions, which are so often buried within ensemble averaging in bulk-scale experiments of biological systems. With the aim of elucidating some of these mechanisms, many studies are designed to observe and manipulate single molecules, such as DNA and proteins either in isolation, or during direct interactions in controlled situations. In the last decades, a wide variety of insightful single-molecule studies have been developed and performed including characterization of molecular motor proteins’ movements^1–8^, direct observation of protein-DNA interactions for various classes of proteins^9–18^, and mechanical characterization of DNA or RNA in various biochemical environments^19–26^.

Single-molecule experimental setups tend to share two essential steps. First, one component (e.g. DNA) must be immobilized onto the surface of a microscope coverslip^10,18^, or bound to a polystyrene microsphere which in turn can be held in an optical or magnetic trap^18,21,27^, or a micropipette^19^. Subsequently, the interaction of the second component (e.g. proteins) with the immobilized molecule, or structural changes in the immobilized molecule caused by exposure to different biochemical environments or mechanical tension, can be observed using advanced light microscopy and force spectrometry techniques^18–23,28,29^. Depending on the aim and the design of the experiment, the configuration of these steps and type of molecules under investigations can vary. However, an essential aspect to all these experiments is the delivery of different substances or solutions to the location at which the immobilized substrate (typically DNA) is being observed, either sequentially or in parallel. To enable this, laminar flow cells (LFCs) with diverse capabilities are designed as essential components of such experiments.

LFCs with a single channel are used when sequential delivery of material to the observation point is needed^10,14,15,18,30^. The inlet of the LFC is connected to a flow pump, which may inject specific media containing the requisite biochemical components consecutively. In these experiments, the DNA substrate is usually immobilized on the surface of the LFC. The challenges with this approach are the need for extensive surface treatment to enable reliable DNA attachment, and the close proximity of the experimental setup to the glass surface, increasing the risk of interference with the biochemical reaction and lowering the accuracy of force spectroscopy experiments. In order to address these challenges and to provide rapid access to different solutions in parallel, multi-channel LFCs have been developed^31–34^. In this type of LFC, two or more physically separated flow channels converge into a common channel in which the experimental observations are made. Due to the low Reynolds number conditions implicit in such setups, and the absence of turbulence in the flow, the contents of adjacent flow streams remain unchanged apart from a small amount of diffusive mixing at the interface. Depending on the design, the widths of each stream tend to vary between 0.2 and 1 mm. Using optical tweezers, trappable components may be rapidly collected in one stream and moved across the interface to a neighboring stream with a different environment. This approach is used extensively to construct DNA dumbbells, which are in turn very useful for both the mechanical characterization of DNA^22^ and the observation of protein-DNA interactions^34^. With this approach, the need for constant flow during the experiment may interfere with protein-DNA interactions and furthermore leads to high consumption of reaction material.

The next important aspect to address with LFCs is the choice of materials and manufacturing methods for the construction of channels (either single or multiple). Typical approaches to fabricating LFCs used in previous studies include (I) cutting the pattern of the channels into a piece of double-sided tape^17,18,30^ or parafilm^23^ and sandwiching it between a coverslip and a microscope slide; (II) engraving channels on the surface of a glass microscope slide^34,35^ or a piece of poly(methyl methacrylate) (PMMA)^36^ using laser etching, hot-embossing or mechanical milling; (III) casting flexible, transparent polydimethylsiloxane (PDMS) material on an inverse silicon master defining the required pattern for the channels^37,38^. A time-consuming and tedious preparation processes, low accuracy and an elevated chance of error in operation, low versatility in design and incompatibility with existing optical setups or targeted biological experiments are common challenges experimentalists face with all three approaches mentioned above. For example experiments with etched channels made by laser or milling machines are limited by poor transparency for experiments using transmission microscopy and force spectroscopy with optical tweezers^34^. Moreover, the tendency of PDMS-made LFCs to shrink and become distorted over time and/or its incompatibility with organic solvents make these surfaces less favorable compared to other types of LFCs^38^. Due to the sensitivity of single-molecule experiments, LFCs are mainly single-use disposable tools. The difficulty of completely cleaning LFCs from any contamination jeopardise subsequent experiments, leading to poor quality results and wasted time and materials. Furthermore commercially available single-use LFCs are costly and limit the possibilities for novel experimental designs. Currently, single-molecule experimentalists are obliged to adjust their experimental plans to be compatible with a limited number of available LFC designs.

**Figure 1:**
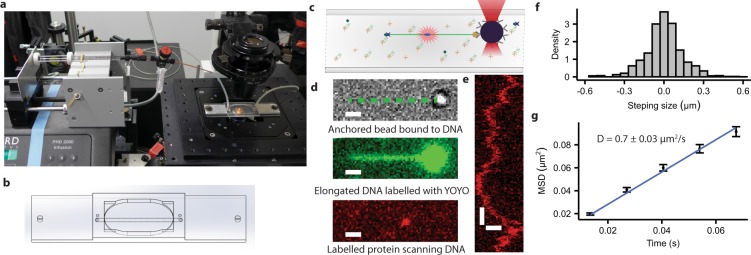
Single-channel LFC. (**a**) Single-channel LFC mounted on the microscope stage and connected to the pumping system. (**b**) The 3D design of the single-channel LFC. (**c**) The depiction of immobilization and linearization of a single DNA molecule (green line) interacting with a fluorescently labelled protein (red circle). The DNA is held in place using an optical trap (red cone) to control a polystyrene microsphere (black circle) attached to one end of the DNA. (**d**) Bright-field microscope image of the trapped polystyrene microsphere (top; position of DNA indicated with dashed line), pseudo-coloured fluorescent image of DNA labelled with YOYO (middle), pseudo-coloured fluorescent image of a single protein molecule labelled with ATTO647N while scanning along the DNA with gamma adjusted to 1.3 (bottom). White scale bars equal 1 µm. (**e**) A kymograph of the movement of protein along DNA with a duration of around 2 seconds; the horizontal and vertical white scale bars equal 1 µm and 200 ms, respectively. (**f**) Density distribution of proteins’ movement within frame intervals of 13.5 m for a collection of 49 trajectories of AlkF as exemplified in (**e**). (**g**) Mean squared displacement (MSD) of 49 scanning trajectories of AlkF along DNA. Error bars represent standard error of the mean (SEM).

In this study, we introduce a new generation of LFCs constructed using additive manufacturing in which the design of the LFC can be easily adjusted to existing optical setups and the requirements of a specific biological experiment (Fig. 1). The rapid and easy process of 3D-design and printing makes the prototyping of LFCs dramatically less tedious and less time-consuming while giving high-accuracy end results and high compatibility with the target single-molecule experiments and optical setups compared to previous approaches. Here we present the design and characterization of a 3D-printed single-channel LFC and perform examples of single-molecule experiments including DNA immobilization, DNA manipulation and protein-DNA interaction. Further, the above-mentioned design-related challenges and limitations of multi-channel LFCs are discussed, and possible solutions are presented. We introduce reservoir-based LFC designs using 3D printing, addressing the challenges related to the general idea of multi-channel LFCs when a flow-free environment is necessary for carrying out biological experiments, and/or separation of nano-scale particles is essential to the experiment. Finally, we demonstrate the construction of a DNA dumbbell using a reservoir-based LFC where the DNA can be moved between different flow-free environments.

## Results and Discussion

### Laminar flow cell production

**Figure 2:**
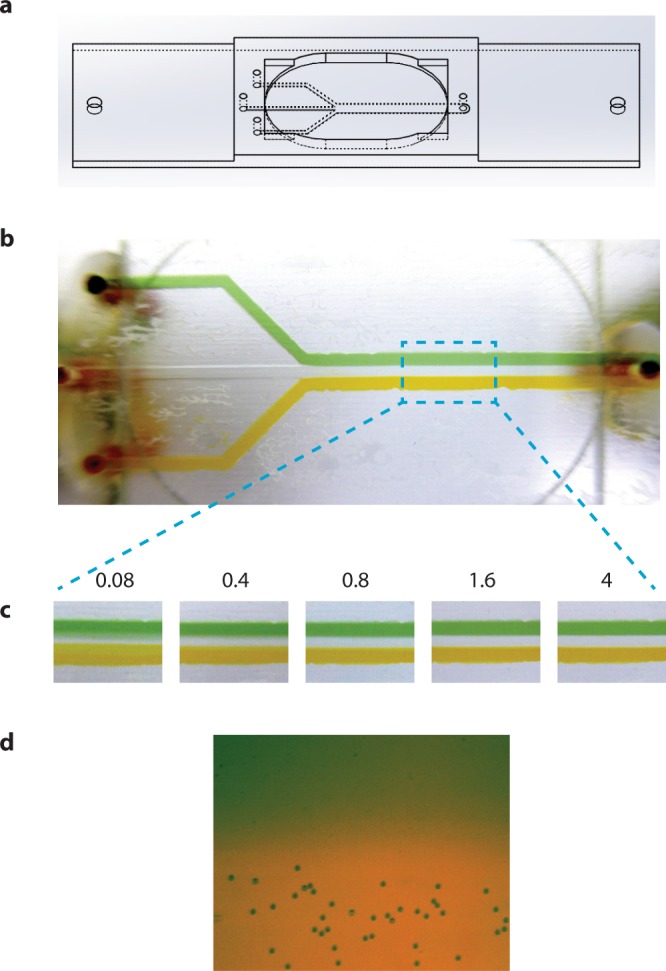
Multi-channel LFC. (**a**) 3D design of a multi-channel LFC. (**b**) Visualization of separation of flow in the multi-channel LFC. The middle channel contains a colour-less buffer solution. (**c**) Quality of separation of flow as a function of flow velocity (mm/s). (**d**) Separation of polystyrene microspheres between two buffer streams running at flow velocity of 0.4 mm/s.

In our approach, the LFCs consist of two parts: a 3D-printed transparent part and a coverslip. The pattern of the channels (approx. 200 µm deep) is imprinted on one side of the 3D-printed component (Figs 1b, 2a and 4b), to which the coverslip is later attached, forming closed channels that are connected to the inlets and outlets. To facilitate the connection of the LFC to the pumping system, PEEK tubes with a length of 1.5–2 cm are attached to the inlets and outlets of the 3D-printed part of the LFC using epoxy glue. With the same epoxy glue, coverslips are attached to the bottom of the 3D-printed component; for this purpose, the glue is applied on a non-absorbing material and spread out evenly as a thin layer over an area as large as a coverslip (around 24 × 60 *mm*). The 3D-printed part of the LFC is placed gently on top of the glue with the side containing the pattern of the channels in direct contact with the glue. A thin layer of glue is thereby deposited evenly across the smooth surface of the 3D-printed component, leaving the channels free of glue. Once the glue is evenly distributed, the 3D-printed part is detached from the surface and a coverslip is attached directly onto it. By applying a light pressure, full contact between the coverslip and the 3D-printed component is ensured, and after a few minutes the LFC can be connected to the pumping system, ready for use.

Based on our experience, the process of constructing a 3D-printed LFC takes much less time (printing of around 10 LFC per hour) than it takes to construct a glass-based or PDMS-based LFC, with a considerably lower rate of errors, such as breakage or leakage. For instance, in the case of glass-based LFCs where double-sided tape is used to attach the coverslip to the microscope slide, and PEEK tubes are directly glued to the microscope slide, we experienced breakage of the glass close to the PEEK-tubes attachment points (possibly due to small cracks in the microscope slide formed during the drilling process) or leakage of the solutions from the coverslip-microscope slide interface after 6 hours of use in around 1/3 of the ca 50 experiments we performed with glass-based LFCs. In the case of our 3D-printed LFCs, no breakage was ever observed, and leakage happened rarely after 6 hours. In addition, depending on the size of the 3D-printed part, the chosen printing technology and selected material, the cost of 3D-printed LFCs is only ~10–30% of currently available commercial LFCs.

The 3D-printed components used in this study are produced with a lateral and axial resolution of around 42 µm and 16 µm, respectively, which is far superior to what can be achieved by alternative methods such as cutting double-sided tape or parafilm manually. The printed material (VeroClear, Stratasys Ltd) is strong and thanks to the flexibility of the design, the LFC can be easily adjusted, stably attached to available microscope stages and connected to the pumping system (Fig. 1a). This addresses the challenge of working with brittle glass LFCs or distortable PDMS-made LFCs, which are not firmly and stably attached to the stage. According to manufacturer’s manuals the material used for printing these LFCs match PMMA in optical properties, which has already proven suitable for different microscopy techniques^36,39^. In a control experiment after 30 min incubation of the surface with polystyrene beads, we did not observe a significant difference in the number of beads nonspecifically bound to the 3D-printed surfaces (in average around 1.3 beads in 0.003 mm^2^ field of view) compared to glass-based surfaces (0.9 beads) or commercial LFCs (1 bead). Resolution of the 3D-printing, and mechanical and optical properties of the LFCs, are mainly defined by the technology of printing and the material used, which are constantly undergoing improvement.

### Single-channel laminar flow cell

Here we show how 3D-printed single-channel LFCs (Fig. 1b and Supplementary File 1) can be used to perform single-molecule experiments involving DNA and proteins. The LFC contains DNA attached to the coverslip via streptavidin-biotin linkers at one end and attached via anti-digoxygenin to a polystyrene bead, which is held in an optical trap, at the other end (Fig. 1c). Once DNA strands are immobilized and attached to the polystyrene microspheres, any unassociated and unwanted component can be washed away from the LFC by running buffer solution through the channel. The anchoring of DNA is verified using bright-field microscopy (Fig. 1d, top panel, and Supplementary Video 1) by observing the behavior of the polystyrene microsphere in the trap. As shown in Supplementary Video 1, the restoring force from the anchored DNA prevents beads from moving freely in a steerable optical trap. By injecting intercalating dye (YOYO) into the LFC, DNA can be visualized by fluorescence microscopy (Fig. 1d, middle panel, and Supplementary Video 2). In a separate experiment, instead of using intercalating dye, fluorescently labelled AlkF protein molecules^40^ are added to the flow cell. The AlkF protein is known to bind to various forms of branched DNA^40^, and here we use it as an example of a DNA scanning protein. The protein is labeled with fluorescent dye ATTO 647N (ATTO-TEC) via maleimide-coupling to a native cysteine in the AlkF (the C-terminal Cys235). After localization of the anchoring point of the DNA, using optical trapping of the DNA-bound bead, the DNA is elongated to around 95 percent of its contour length. The sample is illuminated using the 647 nm laser line in highly inclined illumination mode (HILO)^41^, and the scanning of the protein along DNA is recorded in real time in a flow-free environment (Fig. 1d bottom panel, 1e and Supplementary Video 3). Figure 1e shows a kymograph of the protein’s movement along the DNA for around 2 seconds. A symmetric distribution of stepping size and direction for a combined collection of 49 trajectories of AlkF moving along the DNA (Fig. 1f), implies that the movement of the protein along DNA is unidirectional. In addition, as shown in Fig. 1g, the mean squared displacement (MSD) of the scanning varies linearly with time, which is in accordance with movements of Brownian dynamic. This resonates well with the fact that AlkF does not use any form of biochemical energy (such as ATP) or conformational change for DNA scanning, and that the movement along DNA is thermally driven. From the diffusion equation *D* = 〈*x*^2^〉/2t and data presented in Fig. 1g, the diffusion constant for AlkF is calculated to be 0.7 ± 0.03 µm^2^/s. Many single-molecule studies of protein-DNA interactions are based on analysis of such scanning along the DNA which reveals valuable information about dynamic of these interactions^10,12–14,16,18,42–45^.

### Multi-channel laminar flow cell

To avoid complications due to proximity to the surface and to have rapid access to different types of materials (e.g. small-molecule compounds, proteins, DNA oligonucleotides, coated beads) at the point of observation, multi-channel LFCs have proven useful in single molecule studies. Here, similar to the 3D-printed single-channel LFC described above, the pattern of channels is imprinted on one side of the design (Fig. 2a and Supplementary File 2). The liquids, flowing from physically separated channels, converge into the main channel and continue along the channel in laminar streams with negligible mixing. Figure 2b and Supplementary Video 4 show an experiment with two differently colored solutions on either side and a colorless solution in the middle running through the LFC. The quality and stability of separation of the solutions are dependent on the absence of turbulence in low Reynolds number conditions. The Reynolds number (Re), as a dimensionless criterion for distinguishing laminar flow from turbulence, is calculated from Re = vlρ/η where v is flow velocity, l is the characteristic length of the channel, ρ and η are the density and viscosity of the solution, respectively. The cross-section of the main channel in the 3D-printed multi-channel LFC is 0.2 × 3 mm^2^, and with a typical flow velocity of around 80 μm/s, the Reynolds number is calculated to be around 0.015. This value for the Reynolds number is well below the upper limit for laminar flow (around 2000)^46^ and is in agreement with our observation of separation of adjacent streams in our multi-channel LFC. Injected polystyrene microspheres (diameter ~0.9 µm) follow the same pattern by traveling along the main channel in the same stream as they enter the flow cell, with negligible mixing with the adjacent stream (Fig. 2d and Supplementary Video 5). In Supplementary Video 6 we visualize the translocation of polystyrene microspheres into an adjacent stream using optical tweezers.

The small dye molecules (nm scale) in the colored solutions demand a higher flow speed for clear separation than do the much larger polystyrene microspheres (µm scale). For example, for an average flow velocity below 0.8 mm/s, there is a tendency for the two colored solutions to mix with the middle colorless stream (Fig. 2c). This is due to the fact that smaller particles, such as dyes or other nano-scale chemical compounds in the solution have a much higher diffusion coefficient, resulting in a larger degree of transverse diffusion across the interface between streams in a given period of time. Since slow flows take longer to traverse the length of the experimental chamber, mixing can become a significant issue at slow flow rates. On the other hand, a high flow velocity can disturb the biological interaction under investigation. This indicates that although multi-channel LFCs are good for separation of larger particles at the micro-scale, they might face some limitations when it comes to nano-scale particles such as proteins or fluorescent dyes. Hence, in order to investigate this issue in more detail, we calculated the average displacement of different particles across the streams in the main channel using the concept of transverse diffusion. The mean square transverse displacement of particles across the streams is obtained from the diffusion equation:

1 $$\langle {x}^{2}\rangle =2Dt$$

where *t* is time and the transverse diffusion constant *D* is given by:

2 $$D={k}_{B}T/6\pi \eta a$$

where k_B_, T, η and *a* are Boltzmann’s constant, the temperature in Kelvin, the viscosity of the solution, and the hydrodynamic radius of the particle, respectively. For particles to travel a distance of *l* along the channel with a flow velocity of *v*, it takes time ${\rm{t}}=1/{\rm{v}}$. By combining the expression for time *t* and the diffusion constant *D* from Eq. (2) into the Eq. (1), we can calculate the displacement of particles across the channel (due to thermal diffusion) while they move along the channel, and refer to this as the average transverse displacement (TD_A_):

3 $$T{D}_{A}={({k}_{B}Tl/3\pi \eta av)}^{1/2}$$

**Figure 3:**
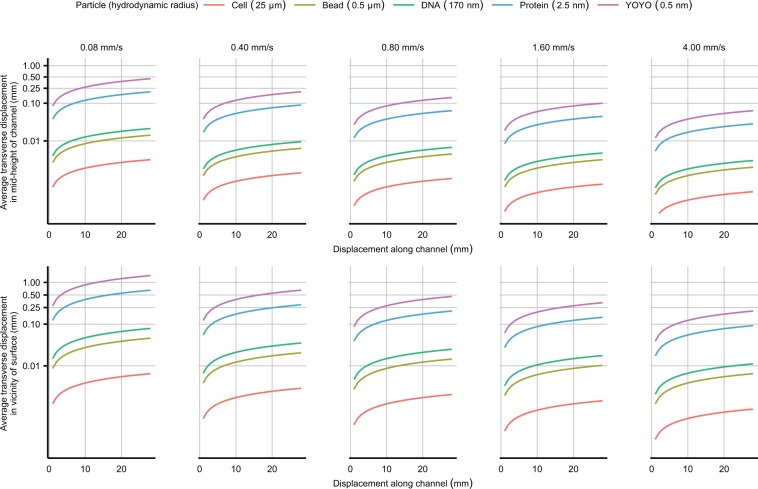
Mixing of materials between streams in a multi-channel LFC. The average transverse displacement of materials with different hydrodynamic radii representative of typical cells, microsphere beads, DNAs, proteins and fluorescent dyes (colored curves) at different flow velocity from 0.08 mm/s (left)–4.00 mm/s (right) are plotted as a function of longitudinal displacement (x-axis) and distance from coverslip surface (upper and lower row). The curves represent the one standard deviation level for a normal distribution of diffusion displacements. The y-axis is plotted with logarithmic scale.

Figure 3 shows the calculated *TD*_*A*_-values for 5 different particles with approximate hydrodynamic radii corresponding to a typical cell, a polystyrene microsphere, an 11 kilobase DNA^47^, a globular protein and a typical fluorescent dye molecule, for different average flow velocities matching those that were used in the experiments. The curves in the plot show the displacement of particles into the adjacent streams at the level of one standard deviation, assuming that the diffusive movement is normally distributed. The closer the layers of fluid are to the upper and lower surfaces of the channel, the more they are subject to shear flow^48^. By looking at a typical flow profile in a square microchannel^49^ it can be inferred that the average flow velocity for the 5 micrometers of fluid closest the flow cell surfaces is around 10% of the flow velocity in the mid-height of the channel. Consequently, transverse displacement and mixing will be more significant close to the surfaces. Therefore, these calculations were performed separately for the case where particles are travelling in the vicinity of the coverslip surface, or at the mid-height of the channel.

In our multi-channel LFCs, the width of the individual streams inside the main channel is around 1 mm. As shown in Fig. 3, the transverse displacement (i.e. mixing of particles into the adjacent stream) for particles with a size of a typical protein or a fluorescent dye in the mid-height of the main channel, is substantial for average flow velocities below 0.8 mm/s. Furthermore, the transverse displacement of these two typical small-sized particles in the vicinity of the surface of the LFC is significant even for a flow velocity of around 4 mm/s. This introduces a serious limitation to the application of multi-channel LFC for smaller particles such as proteins and single fluorescent dyes since they can easily cover a large part of the surface of the LFC and act as a source of noise for fluorescence microscopy. Furthermore, Fig. 3 can be used as a guide for adjusting the design of the LFC and flow rate according to the size of the particles that are used in the experiment.

### Reservoir-based laminar flow cell

**Figure 4:**
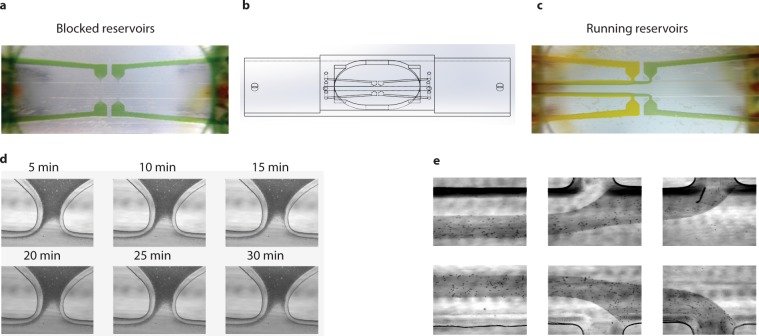
Reservoir-based LFC. (**a**) Visualization of flow-free reservoir-based LFC. (**b**) 3D design of the reservoir-based LFC. (**c**) Visualization of running-reservoirs LFC. (**d**) Stability of materials (polystyrene microspheres) inside the flow-free reservoir at selected time points, with absence of flow in the main channel. (**f**) Visualization of separation of flow in the outlets of the reservoirs and in the main channel in the case of running reservoirs.

Although proven useful when performing several types of single-molecule experiments, the limitations of multi-channel LFCs introduce the need for further improvement in the design and production. The need for constant flow to maintain the separation of materials in multi-channel LFCs can disturb the interaction between molecules under observation; for example it was recently reported that the RecA protein, known for assembling a filament with single-stranded DNA and locating its double-strand homologs, does not interact with DNA under tension caused by either flow or optical trapping^34^. This example of the unexpected lack of a protein-DNA interaction caused by experimental interference introduces the need to perform these experiments in a flow-free environment. Using laser etching of the surface of microscope slides, the same research group designed a flow-free area, called a reservoir, next to the main channel of a multi-channel LFC to face this challenge. The laser etching technique used in that study creates an opaque surface on the microscope slide, which impairs performance of transmission microscopy. Furthermore, in the aforementioned study the multi-channel LFCs with constant running flow is still needed for the separation of material in the flow cell. This introduces serious limitations to separation of smaller particles such as proteins and single fluorescent dyes (Fig. 3) as well as increased amount of material consumed in the experiment due to the need for constant flow. Considering these challenges and limitations to the multi-channel LFCs, and taking advantage of the flexibility of design using 3D-printed LFCs, we developed a reservoir-based LFC. As shown in Fig. 4b and Supplementary File 3, in this design, multiple reservoirs are placed adjacent to the main channel, with each reservoir connected to a pumping system at the inlet. These reservoirs can act as hybrids between flow-free reservoirs to temporarily store valuable and limited materials (Fig. 4a,d) and flow-controlled reservoirs to create downstream laminar-flow channels (Fig. 4c,e).

One advantage of this design over multi-channel LFCs is that the main channel, and particularly the part upstream of the reservoir inlets, remains free of the materials stored inside the reservoirs. Hence, the chance of contamination of the experiment by smaller, fast diffusing particles is lowered significantly if the point of observation is placed upstream of the first pair of reservoirs. Moreover, as shown in Fig. 4a, the flow from the reservoirs can be stopped, resulting in the materials remaining stationary inside the reservoirs with the main channel being mainly free from content of the reservoirs for a relatively long period of time (30 min), in either the absence or presence of flow in the main channel (Fig. 4d). The content of the reservoirs can easily be accessed using optical tweezers (Supplementary Video 7), and the reservoirs can be refilled with fresh material using the pumping system. This LFC design addresses the needs for a flow-free area for experimental observation, combined with the possibility of saving valuable materials while still having convenient access to all required reaction components. Furthermore, this design also allows the reservoirs to act as inlets for separate channels similar to the multi-channel LFCs (Fig. 4c,e and Supplementary Video 8).

**Figure 5:**
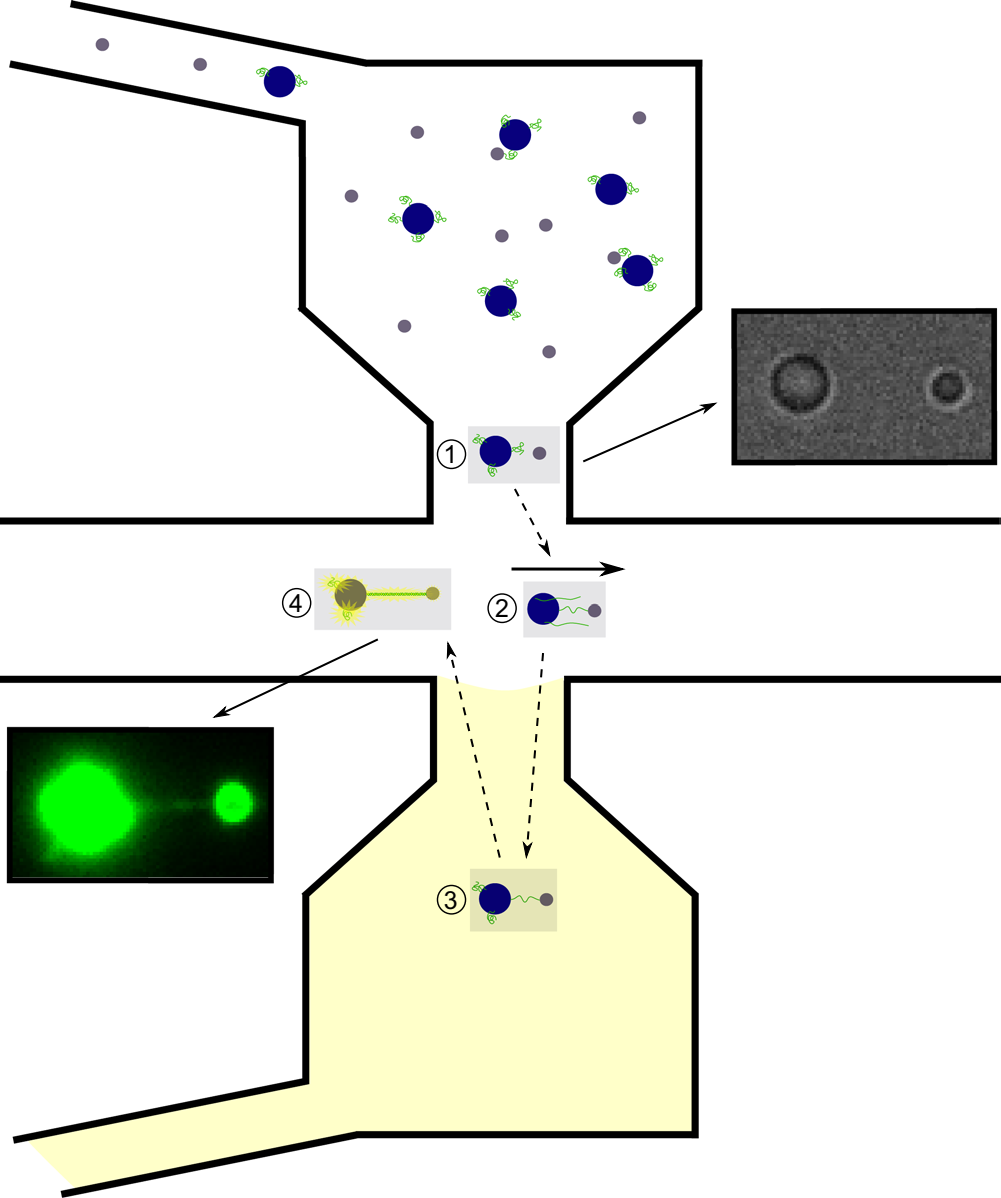
DNA dumbbell construction. Step-by-step process of DNA dumbbell construction and exposure to intercalating dye in a flow-free environment using reservoir-based LFC. (1) Individual trapping of DNA-attached streptavidin coated (diameter: 1.76 µm) and free anti-digoxigenin coated beads (diameter: 0.9 µm) within the reservoir and translocating into the main channel; (top-right inset: bright-field image of the trapped beads) (2) applying gentle flow (0.05 mm/s) for a few seconds to elongate DNA and attachment to second bead (3) Incubation of the DNA dumbbell with YOYO-1 in a flow-free reservoir. (4) Visualization of the DNA in the flow-free main channel; bottom-left inset: fluorescent image of DNA dumbbell.

DNA dumbbells are proven useful for both mechanical characterization of DNA and observation of protein-DNA interactions. In several previous studies multi-channel LFCs were utilized to construct DNA dumbbells and expose them to different biochemical environments^21,22,31,34^, with the separation of these environments being mainly flow-based. Considering the challenges of performing experiments with multi-channel LFCs as discussed in this study, here we use our reservoir-based LFC design to construct a DNA dumbbell and expose it to different biochemical environments without needing high velocity constant flow for separation of the materials. As demonstrated in Fig. 5 and Supplementary Video 9, the components of a DNA dumbbell (DNA and beads) are kept in one reservoir and the DNA intercalating dyes in another reservoir as an example of different environments for controlled exposure of DNA. To attach DNAs to streptavidin-coated polystyrene beads (diameter ~1.76 μm), the beads were initially incubated with digoxigenin-biotin-tagged λ-DNA for one hour. Afterwards the DNA-bead solution was mixed with anti-digoxigenin-coated beads (diameter ~0.9 μm) and injected into one reservoir. Using multiple optical traps, pairs of these beads are moved to the main channel. By applying a gentle flow for a short period of time (around 1 minute) in the main channel the DNA is elongated from the trapped streptavidine-coated bead and the free DNA end can bind to the second bead forming a DNA dumbbell. The rest of the experiment can be performed in a flow-free environment and this addresses the above-mentioned challenges of multi-channel LFCs with regard to the need for constant flow. In the next step, the DNA dumbbell is moved to the opposite reservoir containing the YOYO-1 intercalating dye and after a short incubation, it is moved back to the main channel. The main channel is free of fluorescent dyes allowing for imaging in the absence of noise from any fluorescent particles in the observed volume or non-specifically attached to the surface of the coverslip. This addresses the second limitation of multi-channel LFCs in which nano-scale particles (e.g. proteins and fluorescent dyes) could contaminate the surface of the LFC. DNA dumbbells are constructed in a reservoir-based flow cell and controllably exposed to a different environment without needing constant running flow in the LFC in this exemplary experiment. This approach can be used for multi-component single-molecule experiments where DNA needs to be sequentially exposed to different biochemical environments kept in separate reservoirs (as many as needed, based on the LFC design) before its mechanical or biochemical properties will be characterized either inside that environment or in an uncontaminated observation point in the common channel.

## Conclusion

In this study, we show how the recently developed technology of 3D printing can improve the quality, practicality and customisability of laminar flow-cells used in single-molecule experiments. We also demonstrate that different types of microscopic imaging techniques including bright-field transmission, single-molecule fluorescence detection, and optical trapping are applicable when using 3D-printed LFCs. Moreover, we have shown examples of biological experiments including DNA single-molecule isolation, DNA dumbbell construction and visualization of protein-DNA interactions using these LFCs. We addressed the challenges and limitations of commonly used multi-channel LFCs, such as the need for constant flow and poor separation of nano-scale particles and introduced reservoir-based LFCs as a solution to the challenge. We have discussed important advantages of additive manufacturing compared to previous approaches in production of LFCs for single-molecule studies. This includes versatility and flexibility of the design, which helps fine-tuning the LFCs to the related biological experiment, e.g. by design of multiple, flow-free reservoirs, compatibility with the optical setup, time-efficiency and ease of production and reduced risks of handling errors, as well as improved cost-benefit ratio compared to commercially available options.

## Methods

### Preparation of single-molecule experiment components

#### DNA substrate

The DNA is anchored to the surface of a coverslip at one end and attached to a polystyrene bead at the other end (Fig. 1c) or attached to two different polystyrene beads (Fig. 5). To enable the specific binding of DNA to those surfaces, the substrate was produced with one biotin- and one digoxigenin-containing terminal. This DNA substrate was prepared by PCR amplification of λ-DNA using primers modified with 5′ biotin (5′-bio-ACTTCGCCTTCTTCCCATTT-3′) and 5′ digoxigenin (5′-dig-ATCTCGCTTTCCACTCCAGA-3′) (Eurofins MWG/Operon). The PCR reaction was performed in a 1x LongAmp Taq reaction buffer, with a final volume of 50 μl, 300 µM of each dNTP, 0.4 µM of each primer, 2 units LongAmp Taq DNA polymerase (New England Biolabs) and 0.1 ng/µl of λ-DNA template. The PCR protocol included an initial denaturation step at 94 °C for 3 min, followed by 35 cycles of denaturation (94 °C for 15 s), annealing (60 °C for 60 s) and extension (65 °C for 16 min), followed in the end by a final extension step at 65 °C for 10 min.

#### Bead activation

Carboxylate-modified polystyrene beads with a mean particle size of 0.9 µm (CLB9, Sigma-Aldrich) were coated with an anti-digoxigenin antibody, which can bind to the digoxygenin labelled end of the DNA. 50 µl of the bead stock solution was mixed with 400 µl 50 mM MES buffer (pH 6) and centrifuged at 10,000 *g* for 5 minutes, the supernatant was discarded and the procedure repeated twice. Thereafter, 200 µl of MES buffer supplemented with 50 mg/ml EDC were added to the washed beads, and the mix was incubated and gently shaken for 30 minutes. 200 µl of MES buffer were added, before the beads were centrifuged and finally washed twice with 400 µl 0.1 M borate buffer, pH 8.5. Then 50 µl of a 1 mg/ml anti-digoxigenin antibody solution were added together with beads in 150 µl borate buffer and the mixture was incubated for 4 hours with gentle shaking. Finally, beads were incubated for 2 hours and washed three times with 200 µl 50 mM TRIS buffer, pH 7.5. The coated beads were diluted and stored in a pH 7.5 PBS buffer supplemented with 0.5 mg/ml BSA and 0.1% Tween-20.

#### Protein purification and labeling

The AlkF protein was expressed in BL21-CodonPlus (DE3)-RIPL *E*. *coli* cells (Agilent Technologies) transformed with a pETM-11 (EMBL) plasmid coding for *B*. *cereus* AlkF with an N-terminal hexahistidine tag. The culture was grown in LB broth medium (Gibco) at 37 °C until log-phase, then induced with 0.25 mM IPTG and kept at 18 °C overnight. Cells were harvested by centrifugation at 17000 *g* for 30 min, and 15 ml of sonication buffer (300 mM NaCl, 10 mM imidazole, 50 mM TRIS pH 8.0 and 10 mM 2-mercaptoethanol) per liter of growth medium was added to the cell pellet. A protein extract was prepared by 3 × 30 seconds sonication followed by centrifugation at 27000 *g* for 40 minutes. The protein was purified by IMAC using Ni-NTA agarose (G Biosciences), eluting the protein in sonication buffer supplemented with 50 and 300 mM imidazole. Fractions were analyzed by SDS-PAGE and fractions rich in AlkF were pooled and concentrated using an Amicon Ultra-15 centrifugal filter unit with 10 kDa cut-off filter (Millipore). The buffer was simultaneously replaced by 1x PBS buffer. The AlkF protein was labeled with ATTO 647N (ATTO-TEC) by incubation of AlkF with the dye in a molar ratio of 1:2 for 30 min at room temperature in the dark. The labeling is performed via coupling of a maleimide-modified fluorescent dye ATTO 647N (ATTO-TEC) and a native cysteine in AlkF (C-terminal Cys235). The labelled protein and excess free dye were separated using a NAP-5 column (GE Healthcare). The labelled protein was quantified using a NanoDrop One spectrophotometer (ThermoFisher), and the labelling efficiency was estimated to ~50%. The labelled protein was stored on ice until used for imaging experiments.

### Surface treatment for protein-DNA interaction experiment

As depicted in Fig. 1c, the coverslip surfaces used in the protein-DNA interaction experiments are coated with polyethylene glycol (PEG-NHS, MW = 5000 Da, Nanocs) and biotinylated PEG-NHS (Biotin-PEG-NHS, MW = 5000 Da, Nanocs) in a ratio of 1500:1 to prevent proteins from non-specifically interacting with the glass substrate while also providing binding sites for the biotinylated DNA. Beforehand the coverslips were thoroughly cleaned in a glass staining rack by alternating sonication in 1 M KOH and ethanol (3 times each, for 10 minutes). Next, the coverslips were rinsed with acetone and sonicated for 10 min. In a separate dish, 98 ml of acetone was mixed with 2 ml 3-aminopropyltriethoxysilane (Sigma-Aldrich) in order to functionalize the coverslip glass surface with reactive amine groups for later attachment of amine-linked polyethylene glycol (PEG). The coverslips were incubated in this solution for a total of 5 minutes with a 30 second sonication in the middle of the incubation period. Then, the coverslips were rinsed extensively with water and placed inside an oven and kept at 100 °C for 30 min, followed by cooling to room temperature. 120 µl of a mixture of 150 mg/ml PEG-NHS and 0.1 mg/ml biotin-PEG-NHS in 0.1 M sodium bicarbonate solution were sandwiched between two amino-functionalized coverslips and the slides were kept in a humid chamber overnight. The following day the coverslips were separated, rinsed with water and kept in a vacuum until they were to be used in the construction of the LFC.

### 3D designing and printing

All 3D designs in this study (Figs 1b, 2a and 4b and Supplementary Files 1, 2 and 3) were performed using computer-aided design software (SOLIDWORKS) and the parts were printed using an Objet Connex 500 3D printer (Stratasys Ltd). In order to be able to perform transmission microscopy, we used VeroClear (Stratasys Ltd) material which resembles poly(methyl methacrylate) (PMMA) in optical and mechanical properties. The parts were printed such that the surface containing the channels faced upwards and treated in glossy mode. In glossy mode, the last layer of printed material is thoroughly cured with ultraviolet light (UV) and dissolvable support material is not applied on the top side of the printed part. The thickness of layers was 16 µm and printing was performed at 42 µm resolution. With this configuration, around 10 of these components can be printed in 1 hour, and after removal of supporting material, the parts are ready for construction of the LFCs. The parts are designed to be firmly attached onto the microscope stage, and in order to limit microscopic drift, we used two metal plates for support on either side of the LFC when in place on the microscope stage (Fig. 1a). On a standard inverted microscope stage, the side of the LFC with the coverslip attached is in direct optical contact with the objective lens (usually via a refractive-index matched immersion oil), while the 3D printed part, with a thickness of less than 1 mm, is on the top side and is compatible with the working distance of commonly used condenser lenses (Fig. 1a).

### Laminar flow cell assembly

To assemble the laminar flow cell we started by attaching PEEK tubes with a length of 1.5–2 cm to the inlets and the outlet of the LFC using epoxy glue (UHU SUPER Strong&Safe). In the case of single-channel LFC for construction of the surface-bead-DNA system, the PEGylated coverslips should be taken out of vacuum immediately before use. The surface was coated with streptavidin by sandwiching a 120 µl streptavidin solution (from *Streptomyces avidinii*, Sigma-Aldrich) with a concentration of 0.01 mg/ml in 1x PBS, between one PEGylated coverslip and another clean but not functionalized coverslip for 5 minutes. The coverslips were then carefully separated, rinsed with water and dried with nitrogen gas. Coverslips were attached to the bottom of the 3D-printed component of the LFC, using an all-purpose adhesive (UHU SUPER Strong&Safe) with a very rapid drying time. The process of incubation of coverslips with streptavidin and gluing of the 3D-printed part can be performed in parallel. The construction process is the same for multi-channel or reservoir-based LFCs with differences in surface treatment of the coverslip depending on the precise requirements of the biological experiment.

To achieve homogenous and airtight attachment and avoid filling the channels with glue, we used stamping techniques similar to those utilized in lithography. The glue was applied on a non-absorbing material and spread out evenly by applying even pressure using the edge of a glass coverslip. With the glue evenly distributed over an area as large as a coverslip (around 24 × 60 *mm*) the 3D-printed part of the LFC was placed gently on top of the glue with the side containing the pattern of the channels in direct contact with the glue. A thin layer of glue is thereby deposited evenly across the smooth surface of the 3D-printed component, leaving the channels free of glue due to the physical separation between the two surfaces. Other than the channels, the remainder of the surface should be covered with glue, and gentle pressure can be applied to the points where the surface is not covered. To avoid penetration of the glue into the channels the 3D-printed part was detached from the surface carefully without any horizontal movement. The streptavidin-coated coverslip was then placed directly onto the 3D-printed part and full contact was ensured by applying light pressure where necessary on the surface (using a soft plastic object such as pipette tip). Special care should be taken to ensure that the glue is well distributed at the edges of the channels, reservoirs, inlet(s) and outlet. After a few minutes of drying, the LFC is ready to be connected to a pumping system (e.g. Harvard Apparatus PHD 2000).

### Surface-DNA-bead and DNA dumbbell constructions

For construction of the surface-DNA-bead complex used in this study, the single-channel LFC containing the streptavidin-coated coverslip was incubated with blocking buffer (25 mM Tris-HCl, pH 7.5, 2 mM EDTA, 1–3 mg/ml BSA, 0.01% (v/v) Tween-20) for around 1 hour, followed by 20 min incubation with a 25 mM TRIS, pH 7.5 buffer with 10 ng/ml DNA, then a washing step using the TRIS buffer, and finally 1 hour incubation with polystyrene microbeads dissolved in a 7:3 mix of TRIS and blocking buffer. Three-way valves (V100T, Upchurch Scientific) were used in order to switch the flow between a supply tank of different solutions and the LFCs.

To construct DNA dumbbells, a reservoir-based LFC was initially incubated with a solution of 5 mg/ml BSA for 1 hour to saturate the surface with protein and prevent non-specific binding to the coverslip surface. Streptavidin-coated beads (diameter ~1.76 µm, Spherotech) with concentration of 0.01 mg/ml were incubated with a digoxigenin-biotin-tagged 12 kb fragment of λ-DNA with a concentration of 0.4 ng/μl in 500 μl PBS buffer for 1 hour. This solution was mixed with anti-digoxigenin-coated beads (diameter ~0.9 µm) with a final concentration of 0.01 mg/ml in PBS buffer supplemented with 1 mg/ml BSA and injected into one of the reservoirs. The other reservoir was filled with YOYO-1 intercalating dye (ThermoFisher Scientific) dissolved in a ROXS buffer consisting of 400 μl of 10% w/v glucose, 34 μl of 60 mM ascorbic acid (Fluka), 34 μl of 98% 60 mM methylviologen hydrat (Sigma-Aldrich), 10 μl of 1 M TRIS at pH 8, 2 μl of 10 μM YOYO-1, 50 μl of enzyme stock solution and 480 μl double-distilled water. The enzyme stock solution was prepared separately by mixing 1 mg glucose oxidase (Sigma-Aldrich), 6 μl of 45 mg/ml catalase (Sigma-Aldrich), 50 μl PBS and 950 μl double-distilled water.

### Optical setup

LFCs were secured on a piezo-steerable stage on an inverted microscope (IX-7; Olympus). Super-resolution imaging and holographic optical trapping were performed through combined custom-build setups, which have been described before^50^. Briefly, fluorescent illumination was conducted using an argon-krypton ion laser (Innova 70C; Coherent) with the laser lines 488 and 647 nm. The 488 nm laser line was used to image DNA stained with an intercalating dye YOYO-1 (ThermoFisher Scientific) and the 647 nm laser line was used to observe proteins labelled with ATTO 647N (ATTO-TEC). The fluorescent laser beam was focused on the back focal plane of the microscope objective lens (PlanApo N, x60 NA 1.49; Olympus). A HILO (highly inclined and laminated optical sheet)^41^ illumination method was employed to decrease background noise outside the focal plane, thereby achieving a superior signal to noise ratio. To perform HILO the beam was steered at the back focal plane of the objective lens using an adjustable mirror. Fluorescence emission and bright-field images were projected onto an EMCCD camera (iXon DV887DCS-BV; Andor) with a scale of 112 nm per pixel.

For holographic optical trapping, a 2W diode-pumped solid-state infrared laser (MIL-H-1064; CNI) emitting a wavelength of 1064 nm was used. Multiple-steerable trapping were achieved using a spatial light modulator (SLM) (XY Series 512 × 512; Boulder Nonlinear Systems). The SLM was imaged onto the back focal plane of the same microscope objective lens mentioned before by a 4f-telescope. To allow simultaneously optical trapping and fluorescence imaging, the trapping light and the fluorescent excitation light were overlaid and coupled into the microscope light path using a dichroic mirror (NFD01-1064-25x36, Semrock).

For data acquisition, the EMCCD camera was controlled by open source software Micro-Manager^51^. Pictures and videos of the multi-channel laminar flow cell and the reservoir based flow cell were taken using a digital camera (Panasonic Lumix DMC-LF1) as well as a colour CMOS camera (UI-1240SE-C-GL CMOS; IDS) for brightfield imaging. In addition to the 60x magnification objective lens, which was used for trapping and fluorescence imaging, images also were acquired using a 20x magnification objective lens (UCPlanFL N, x20 NA 0.70; Olympus) and a 4x magnification objective lens (Plan N, x4 NA 0.10 Olympus) to provide a wider field of view.

## Supplementary information

Supplementary information

Supplementary_video1

Supplementary_video2

Supplementary_video3

Supplementary_video4

Supplementary_video5

Supplementary_video6

Supplementary_video7

Supplementary_video8

Supplementary_video9

Supplementary_file1-3

## Data Availability

All data generated or analysed during this study are available from the corresponding author on reasonable request.
