# Supplementary material for: Additive manufacturing of laminar flow cells for single-molecule experiments
Source: Sci Rep. 2019 Nov 14;9:16784. doi: 10.1038/s41598-019-53151-z (PMC6856346; doi:10.1038/s41598-019-53151-z)
Supplement: Supplementary file 1 — Supplementary information [file 41598_2019_53151_MOESM1_ESM.pdf]

Additive manufacturing of laminar flow cells for single-molecule experiments (supplementary information)

Arash Ahmadi[1], Katharina Till[2], Yngve Hafting[3], Mark Schüttpelz[2], Magnar Bjørås[4,5], Kyrre Glette[3], Jim Tørresen[3], Alexander D. Rowe[1,6,*], Bjørn Dalhus[1,5,*]

[1] Department of Medical Biochemistry, Institute for Clinical Medicine, University of Oslo, Oslo, Norway

[2] Biomolecular Photonics, Department of Physics, University of Bielefeld, Bielefeld, Germany

[3] Department of Informatics, University of Oslo, Oslo, Norway

[4] Department of Clinical and Molecular Medicine, Faculty of Medicine and Health Sciences, Norwegian University of Science and Technology (NTNU), Trondheim, Norway

[5] Department of Microbiology, Oslo University Hospital HF, Rikshospitalet and University of Oslo, Oslo, Norway

[6] Department of Newborn Screening, Division of Child and Adolescent Medicine, Oslo University Hospital, Oslo, Norway

* Corresponding authors

Supplementary file descriptions:

Supplementary video 1: Manipulation of a single DNA anchored to the surface using optical tweezer. A single DNA molecule is anchored to the surface of a coverslip at one end and attached to a polystyrene microsphere at the other end. The microsphere is moved around using the optical trap and the movement is limited within the length of DNA, and the bead falls out of the trap due to resistance force by DNA. This video is an example of applicability of single-channel 3D-printed LFC for bright-field transmission microscopy and optical trapping.

Supplementary video 2: Manipulation of a single anchored DNA in fluorescence microscopy. DNA is labelled with YOYO and the manipulation is visualized using fluorescence microscopy in an experiment with single-channel 3D-printed LFC.

Supplementary video 3: Protein-DNA interaction. Using single-channel LFC, a single protein (fluorescently labelled) is detected while scanning along a linearized DNA in an optical trap. The video is played at half speed and gamma adjusted to 1.3.

Supplementary video 4: Separation of flow into different streams in multi-channel 3D-printed LFC. The liquids flowing from physically separated channels (colored solutions on the sides and clear solution in the middle channel) converge into the main channel and continue along separated streams. By stopping and restarting the flow in the middle channel it is visualized how the stream pattern change. The flow rate is around 50 $\mu l/min$.

Supplementary video 5: Separation of micro-scale beads in multi-channel 3D-printed LFC. The polystyrene microspheres are traveling along the separated streams with negligible mixing. Flow rate is changing from 50 to 10 $\mu l/min$.

Supplementary video 6: Translocating beads across the streams in a multi-channel 3D-printed LFC. The material translocation across the streams is visualized. The polystyrene microsphere is trapped in the stream containing beads and moved to the adjacent stream and released there.

Supplementary video 7: Translocating beads between the reservoirs in reservoir-based 3D-printed LFC. The reservoirs contain polystyrene microspheres and the flow is stopped. The microspheres remain stationary inside the reservoir, and here the translocation of beads from one reservoir to the opposite reservoir using optical tweezers is visualized.

Supplementary video 8: Running flow reservoir-based 3D-printed LFC. The reservoirs act as running flow channels to create separation of different streams inside the main channel. The area upstream of the outlet of the reservoirs remains free of both dyes and microspheres.

Supplementary video 9: Construction of DNA dumbbell. The reservoir-based 3D-printed LFC is used to construct DNA dumbbells and visualize them by optical trapping and fluorescence microscopy in a flow-free environment.

Supplementary files 1,2 and 3: The 3D designs of different versions of 3D-printed LFCs. These files contain the 3D sketches of the single-channel, multi-channel and reservoir based flow cells and are designed using SOLIDWORKS.
